# Trends in Serum Amylase Levels in People Living with HIV: A Comparison Between INSTI and NNRTI/PI-Based Regimens

**DOI:** 10.3390/v18010045

**Published:** 2025-12-27

**Authors:** Elena Rabinovich, Ramon Cohen, Shay Nemet, Haitham Abu Khadija, Shira Bezalel-Rosenberg, Ilan Asher, Keren Mahlab-Guri, Daniel Elbirt

**Affiliations:** 1 Department of Clinical Immunology, Allergy and AIDS, Kaplan Medical Center, Rehovot 76100, Israel; 2 Faculty of Medicine, Hebrew University of Jerusalem, Jerusalem 91120, Israel; 3 Department of Internal Medicine B, Kaplan Medical Center, Rehovot 76100, Israel; 4 Department of Cardiology, Kaplan Medical Center, Rehovot 76100, Israel

**Keywords:** HIV, amylase, NNRTI, INSTI

## Abstract

**Background**: Antiretroviral therapy (ART) has transformed HIV into a chronic manageable condition, yet metabolic toxicities including pancreatic enzyme alterations remain concerns. While older ART regimens have been associated with hyperamylasemia, the impact of integrase strand transfer inhibitor (INSTI)-based therapies on serum amylase levels has not been specifically examined. **Purpose**: This study aimed to compare longitudinal patterns of serum amylase levels between people living with HIV receiving INSTI-based versus NNRTI/PI-based ART regimens. **Methods**: This retrospective observational study analyzed 99 HIV-positive patients at Kaplan Medical Centre, Israel (2002–2023). Participants received either INSTI-based (*n* = 49) or NNRTI/PI-based (*n* = 50) regimens for ≥24 months. Serum amylase, viral load, CD4 counts, and metabolic parameters were measured at baseline, one year, and two years. Repeated-measures ANOVA assessed longitudinal changes. **Results**: NNRTI/PI-treated patients maintained significantly higher mean amylase levels throughout follow-up (baseline: 122.9 ± 42.1 U/L; two years: 129.6 ± 38.0 U/L) compared to INSTI-treated patients (baseline: 78.7 ± 32.3 U/L; two years: 68.4 ± 23.4 U/L; *p* < 0.0001 at all timepoints). A significant linear time-by-group interaction (*p* = 0.037) demonstrated divergent trajectories. No clinical pancreatitis was observed in either treatment group during the follow-up period, and all observed variations in serum amylase were biochemical and asymptomatic. While these findings are reassuring regarding acute pancreatic toxicity, the clinical significance of chronic subclinical enzyme elevations remains uncertain. **Conclusion**: INSTI-based antiretroviral regimens suggest a favorable pancreatic and metabolic safety profile compared with NNRTI/PI-based therapies.

## 1. Introduction

Human Immunodeficiency Virus (HIV) continues to be a significant global health challenge, with millions affected worldwide [1]. The introduction of antiretroviral therapy (ART) has changed the course of HIV infection, transforming it into a chronic, manageable condition markedly improving life expectancy. However, the chronic use of ART has been increasingly associated with metabolic and organ-specific toxicities, including those involving the pancreas [2,3,4]. The pancreas plays an important role in digestion and glucose regulation through the secretion of enzymes such as amylase and lipase. Serum amylase, primarily produced by the pancreas and salivary glands, serves as a key marker of pancreatic function [5]. While elevated serum amylase is associated with acute pancreatitis, among people living with HIV (PLWH), enzyme abnormalities often occur in the absence of clinical pancreatitis [5]. The mechanisms proposed for pancreatic injury in HIV include direct viral effects, opportunistic infections, and drug-induced toxicity associated with antiretroviral therapy [2,3,6,7].

During the pre-Highly Active Antiretroviral Therapy (HAART) era, when mono- or dual-drug antiretroviral regimens were used, pancreatic abnormalities were frequently reported, including asymptomatic hyperamylasemia and a higher incidence of pancreatitis than in the general population [8,9]. With the introduction of HAART in the mid-1990s, combination regimens dramatically improved HIV outcomes but also introduced new treatment-related metabolic disturbances that could secondarily affect pancreatic function [3,4,9,10].

While previous studies have primarily focused on ART regimens containing nucleoside reverse transcriptase inhibitors (NRTIs) combined with non-nucleoside reverse transcriptase inhibitors (NNRTIs) or protease inhibitors (PIs), the effects of integrase strand transfer inhibitor (INSTI)-based therapies on serum amylase levels have not been systematically examined. Recent comprehensive reviews of INSTI safety have focused predominantly on weight gain, dyslipidemia, and neuropsychiatric effects, while longitudinal assessment of pancreatic enzyme dynamics remains underexplored. Given that INSTIs have now become the preferred backbone of first-line antiretroviral therapy worldwide, understanding their long-term pancreatic safety profile through comparison with older-generation regimens represents an important knowledge gap. Our study addresses this gap by examining the differential patterns in serum amylase levels in HIV patients treated with INSTI-based regimens compared to those receiving NNRTI/PI-based regimens over a two-year follow-up period.

To our knowledge, previous studies mostly focused on ART regimens containing nucleoside and non-nucleoside reverse transcriptase inhibitors (NRTIs/NNRTIs) or protease inhibitors (PI), while the effects of integrase strand transfer inhibitor (INSTI)-based therapies on serum amylase levels have not been specifically examined. Our study addresses this gap by examining the differential patterns in serum amylase levels in HIV patients treated with INSTI-based regimens compared to those receiving NNRTI/PI-based regimens.

## 2. Materials and Methods

### 2.1. Study Design and Population

This retrospective observational study analyzed the medical records of people living with HIV who were followed and treated at the HIV clinic of Kaplan Medical Centre, Israel, between 2002 and 2023.

Patients were eligible for inclusion if they had a confirmed diagnosis of HIV, received continuous antiretroviral therapy (ART) of the same regimen class—either PI/NNRTIbased or INSTI-based—for at least 24 months, and had available serum amylase measurements at baseline and during follow-up.

Patients were excluded if they had severe hepatic or renal disease at baseline that could independently affect pancreatic enzyme metabolism or interpretation of serum amylase levels. Severe hepatic disease was defined as follows: (1) documented cirrhosis (ICD-10 codes K74.0-K74.69) or acute liver failure (ICD-10 codes K72.0-K72.9); (2) aspartate aminotransferase (AST) or alanine aminotransferase (ALT) levels > 10 times the upper limit of normal (ULN) at baseline (>400 U/L for AST, >410 U/L for ALT based on our laboratory reference ranges); or (3) total bilirubin > 5 mg/dL with evidence of hepatic synthetic dysfunction. Severe renal disease was defined as follows: (1) end-stage renal disease requiring dialysis (ICD-10 codes N18.6, Z99.2); (2) estimated glomerular filtration rate (eGFR) < 15 mL/min/1.73 m^2^ at baseline; or (3) serum creatinine > 4 mg/dL in the absence of acute reversible causes. These thresholds were selected to exclude patients with organ dysfunction severe enough to substantially alter pancreatic enzyme production, metabolism, or clearance while retaining patients with mild-to-moderate organ impairment that commonly occurs in HIV-infected populations. Laboratory values were excluded as implausible or erroneous if they met any of the following criteria: (1) serum amylase values > 1000 U/L (>10 times the upper limit of normal) without documented acute pancreatitis or clear clinical correlation, as such extreme elevations in asymptomatic patients likely represent laboratory error or specimen handling issues; (2) serum creatinine values > 20 mg/dL without documented acute kidney injury or dialysis requirement, suggesting pre-analytical error; (3) CD4 count or viral load values that were flagged by the laboratory information system as technically invalid due to specimen quality issues; (4) multiple consecutive laboratory values for the same parameter that showed physiologically impossible fluctuations (e.g., CD4 count changing by >500 cells/μL between consecutive measurements one month apart in a stable patient on effective ART) suggesting data entry or laboratory processing errors. All excluded values were reviewed by two investigators independently, and decisions to exclude were documented with rationale in the study database.

Comorbidities were systematically identified through comprehensive review of the electronic medical record system. For each patient, we searched the complete medical record, including (1) the problem list and coded diagnoses using both ICD-9 and ICD-10 diagnostic codes; (2) infectious disease consultation notes and HIV clinic encounter documentation; (3) hospitalization discharge summaries; and (4) subspecialty consultation notes from gastroenterology, endocrinology, hepatology, and nephrology when available. Specific comorbidities relevant to serum amylase interpretation were specifically searched for, including salivary gland disorders (ICD-10: K11.x codes, search terms: “parotitis,” “sialadenitis,” “salivary gland”), gastrointestinal disease (ICD-10: K25–K28 for peptic ulcer disease, K50–K51 for inflammatory bowel disease, search terms: “IBD,” “Crohn’s,” “ulcerative colitis,” “peptic ulcer”), hepatic disease (ICD-10: K70–K77, B18-B19 for viral hepatitis, search terms: “hepatitis,” “cirrhosis,” “fatty liver,” “NAFLD”), renal disease (ICD-10: N18–N19, search terms: “chronic kidney disease,” “renal insufficiency”), diabetes mellitus (ICD-10: E10–E11, search terms: “diabetes,” with confirmation through documented use of antidiabetic medications or repeated fasting glucose > 126 mg/dL or HbA1c > 6.5%), gallbladder disease (ICD-10: K80-K82, search terms: “cholelithiasis,” “cholecystitis,” “gallstones”), alcohol use disorder (ICD-10: F10.x, documentation of alcohol counseling or treatment programs, reported alcohol consumption > 14 drinks/week for men or >7 drinks/week for women), and tobacco use (search terms: “smoking,” “tobacco,” documentation in social history sections, or coded diagnosis Z72.0). All comorbidity data were abstracted independently by two trained reviewers, with discrepancies resolved through discussion and senior investigator review.

Due to the evolution of antiretroviral therapy guidelines and drug availability over the study period, patients in the two treatment groups were enrolled during different time periods. The NNRTI/PI-based regimen group predominantly includes patients who initiated treatment between 2002 and 2015, when these drug classes represented the standard of care, while the INSTI-based regimen group consists primarily of patients who initiated treatment between 2016 and 2023, following the introduction and subsequent widespread adoption of integrase inhibitors as preferred first-line therapy. This temporal separation reflects real-world changes in HIV treatment practices but introduces potential temporal bias, as patients treated in different eras may have experienced differences in overall clinical care, diagnostic protocols, comorbidity management, and patient population characteristics. This limitation is acknowledged and discussed in detail in the Limitations section. The complete study population and exclusions can be found in the supplement.

### 2.2. Data Collection

Demographic and clinical data were extracted from the electronic medical records of 1202 patients treated in the clinic. The collected variables included age, sex, nationality, HIV acquisition risk group, date of ART initiation, and type of regimen. Laboratory parameters included serum amylase, viral load (HIV RNA), CD4 cell count, AST, ALT, and serum creatinine, which were recorded at three time points: baseline (prior to ART initiation), one year, and two years after initiation.

Comorbidities and other medical conditions known to potentially cause mild, non-pancreatic elevations in serum amylase—such as salivary gland disorders, mild hepatic or gastrointestinal disease, diabetes mellitus, or alcohol use—were also reviewed to identify possible confounding factors.

After applying inclusion and exclusion criteria, 99 patients met eligibility requirements and were stratified into two groups according to ART regimen class: Group 1—INSTI-based regimens (*n* = 49) and Group 2—NNRTI/PI-based regimens (*n* = 50). The study was approved by the institutional Helsinki Ethics Committee.

Statistical data analysis was performed using IBM SPSS Statistics software (version 29.0, IBM Corp., Armonk, NY, USA).

Continuous variables were tested for normality using the Kolmogorov–Smirnov test and are presented as mean ± standard deviation (SD) or median with interquartile range (IQR), as appropriate. Categorical variables are expressed as frequencies and percentages. Comparisons between treatment groups (INSTI vs. NNRTI/PI) were performed using independent samples *t*-tests for normally distributed continuous variables and the Mann–Whitney U test for non-normally distributed variables.

Chi-square or Fisher’s exact tests were used for categorical variables, depending on cell counts. Normality of continuous variables was assessed using the Kolmogorov–Smirnov test.

Outlier detection and handling: Extreme values were identified using the interquartile range (IQR) method (values > Q3 + 3 × IQR or <Q1 − 3 × IQR) and clinical review. The large standard deviation observed in creatinine values for the NNRTI/PI group (Table 5) was primarily driven by 2–3 patients with moderately elevated creatinine values (1.4–2.5 mg/dL) due to documented pre-existing mild chronic kidney disease that did not meet our exclusion criteria for severe renal disease (creatinine > 4 mg/dL or eGFR < 15). These values were clinically valid and were retained in the analysis as they represent the real-world patient population receiving ART. Statistical analyses were conducted on raw data without winsorization. Sensitivity analyses were performed excluding the highest creatinine values, which did not materially change the interpretation of results regarding amylase trends.

Multiple comparison considerations: Our primary outcome of interest was serum amylase, with viral load, CD4 count, liver enzymes, and creatinine serving as secondary parameters to characterize the cohorts and assess potential confounding. Given the exploratory nature of this retrospective observational study and the pre-specification of amylase as the primary outcome, we did not apply formal multiple comparison corrections (e.g., Bonferroni). However, we acknowledge that the multiple statistical tests that were performed (across Table 2, Table 3, Table 4 and Table 5) increase the risk of Type I error, and findings for secondary parameters should be interpreted with appropriate caution. *p*-values are reported without adjustment, and interpretation focuses on the consistency and pattern of findings rather than isolated statistically significant results.

The between-subjects factor (ART regimen class) allows assessment of overall differences in outcome levels between treatment groups, while the time × treatment group interaction specifically evaluates whether the temporal trajectory of change differs between regimens, independent of baseline values. This analytical framework is appropriate for addressing our primary research question regarding differential metabolic effects of INSTI-based versus NNRTI/PI-based therapy in clinical practice. To evaluate longitudinal changes in laboratory parameters over time (baseline, 1 year, and 2 years), a repeated-measures ANOVA was conducted, with ART regimen (INSTI vs. NNRTI/PI) as the between-subjects factor and time as the within-subjects factor.

Interaction effects (time × treatment group) were examined to assess differences in trends between regimens.

Post hoc power analysis: Based on our observed sample size (*n* = 99, with *n* = 49 in INSTI group and *n* = 50 in NNRTI/PI group) and the observed effect size for the primary outcome (mean difference in amylase at 2 years of 61.16 U/L with pooled SD of approximately 32 U/L, yielding Cohen’s d ≈ 1.91), our study achieved >99% statistical power to detect the observed difference in amylase levels between groups at α = 0.05 (two-tailed). For the time × treatment group interaction in the repeated-measures ANOVA (our key analysis), with observed partial eta-squared of 0.044 for the linear interaction, the achieved power was approximately 80% at α = 0.05. These power estimates confirm that our sample size was adequate for detecting large between-group differences in amylase levels, though the study would be underpowered to detect small or moderate effects or to conduct multiple subgroup analyses.

Where overall effects were significant, post hoc pairwise contrasts were applied to determine specific differences between time points.

Correlations between continuous variables such as CD4 cell count and viral load were analyzed using Pearson’s correlation coefficients (for normally distributed data). A *p*-value of <0.05 was considered statistically significant for all analyses.

## 3. Results

### 3.1. Overview of the Study Population

A total of 99 patients met the predefined inclusion criteria and were included in this study.

All participants were HIV-positive individuals under regular follow-up.

Patients were divided into two groups according to their initial ART regimen: 49 patients received INSTI-based therapy, and 50 patients were treated with NNRTI/PI-based regimens. After data collection from medical records, a comprehensive statistical analysis was performed, including comparison of demographic and clinical characteristics between the two treatment groups, as well as evaluation of continuous and categorical laboratory parameters.

### 3.2. Demographic and Clinical Characteristics

Most participants were males, and gender proportions were similar between treatment groups (*p* = 0.06). The mean age at treatment initiation was 37.7 ± 12.3 years in the INSTI group and 42.0 ± 16.3 years in the NNRTI/PI group (*p* = 0.136, Table 2).

Ethnic distribution differed significantly between the groups (*p* < 0.001): Ethiopian-origin patients were more prevalent in the NNRTI/PI group (≈60%), whereas Israeli-born and ex-Soviet-origin patients were more frequently represented in the INSTI group (Table 1). After one year of treatment, all patients maintained virologic suppression (<200 copies/mL). No significant baseline differences were observed in viral load and CD4 count (*p* > 0.05), except for creatinine, which showed a statistically significant difference due to extreme variability in the NNRTI/PI group (*p* < 0.0001), although this difference was not clinically meaningful. No significant differences were found in other comorbidities that could potentially influence serum amylase levels (*p* = 0.366, Table 1).

**Table 1:** Baseline demographic and clinical characteristics of study participants stratified by antiretroviral therapy regimen (INSTI vs. NNRTI/PI-based).

		GROUPS			
		INSTI	NNRTI + PI	Total	*p*-Value
	Total	49	50	99	
SEX	M	38 (77.6%)	30 (60.0%)	68 (68.7%)	0.06
	F	11 (22.4)	20 (40%)	31 (31.3%)	
ORIGIN	ETHIOPIA	4 (8.2%)	30 (60.0%)	34 (34.3%)	<0.001
	ISRAEL	21 (42.9%)	10 (20%)	31 (31.3%)	
	EX SOVIET UNION	19 (38.8%)	3 (6.0%)	22 (22.2%)	
	OTHER	5 (10.2%)	7 (14.0%)	12 (12.1%)	
RISK GROUP	END-GEO	3 (6.1%)	33 (66.0%)	36 (36.4%)	<0.001
	MSM	17 (34.7%)	6 (12.0%)	23 (23.2%)	
	IVDU	4 (8.2%)	2 (4.0%)	6 (6.1%)	
	OTHER	25 (51.0%)	9 (18.0%)	34 (34.3%)	
VL ONE YEAR AFTER TREAT	ND+ < 50	46 (93.9%)	48 (96.0%)	94 (94.9%)	0.412
	50–200	3 (2.97)	2 (1.98)	5 (4.95%)	
OTHER COMORBIDITIES THAT COULD RAISE AMYLASE LEVELS	NO	42 (85.7%)	39 (78%)	81 (81.8%)	0.36
	RENAL DISEASE	0	0	0	
	GI (IBD, PEPTIC ULCER)	0	1 (2.0%)	1 (1%)	
	ALCOHOL	1 (2.0%)	2 (4%)	3 (3%)	
	DIABETES	0	3 (6%)	3 (3%)	
	GALLBLADDER DISEASE (Cholelithiasis)	2 (4.1%)	3 (6%)	5 (5.1%)	
	Liver disease (HCV, FATTY LIVER ETC)	3 (6.1%)	1 (2%)	4 (3.96%)	
	SALIVARY GLAND DISORDERS (Parotitis)	0	1 (2%)	1 (1.0%)	
SMOKING	YES	18 (36.7%)	10 (20%)	28 (28.3%)	0.055
	NO	31 (63.3%)	40 (80%)	71 (71.7%)	

Data presented as *n* (%) for categorical variables. Chi-square tests or Fisher’s exact tests were used to compare categorical variables between groups as appropriate. *p* < 0.05 was considered statistically significant. Abbreviations: INSTI, integrase strand transfer inhibitor; NNRTI, non-nucleoside reverse transcriptase inhibitors; PI, protease inhibitor; END-GEO, endemic geographical region; MSM, men who have sex with men; IVDU, intravenous drug use; VL, viral load; ND, not detectable; HCV, hepatitis C virus; IBD, inflammatory bowel disease.

### 3.3. Laboratory and Clinical Parameters

The overall immunologic and virologic profiles were similar across treatment groups, consistent with effective viral suppression and immune recovery (Table 2): At baseline, viral load (VL) and CD4^+^ T-cell counts did not differ significantly between the groups. A significant negative correlation was observed between VL and CD4 count (r = –0.33, *p* = 0.02), indicating that higher viral loads were associated with lower CD4 levels. Liver enzymes (ALT, AST) were comparable between groups, with no statistically significant differences (Table 4). Renal function, as reflected by serum creatinine values, showed higher variability in the NNRTI/PI group at baseline (*p* < 0.0001) but remained stable throughout follow-up (Table 5). Mean creatinine values in both groups remained within the normal range during the two-year period, confirming preserved renal function and excluding kidney impairment as a confounding factor for amylase changes. Serum amylase levels were significantly higher in the NNRTI/PI group at all time points (*p* < 0.0001, Table 3). Patients receiving INSTI-based regimens demonstrated a mild, non-significant decline in mean amylase values over time (from 78.7 ± 32.3 to 68.4 ± 23.4 U/L), whereas those treated with NNRTI/PI-based regimens maintained persistently elevated mean levels (from 122.9 ± 42.1 to 129.6 ± 38.0 U/L) across the two-year follow-up. No cases of clinical pancreatitis were identified in either group during the follow -up. These findings suggest potential differential effects of ART class on pancreatic enzyme

**Table 2:** Baseline characteristics: age, viral load, and CD4^+^ T-cell count at treatment initiation.

Variable	INSTI (*n* = 49)	NNRTI/PI (*n* = 50)	Total (*n* = 99)	*p*-Value
Age (years), mean ± SD/median	37.67 ± 12.26/37	42.04 ± 16.33/41	39.87 ± 14.55/37	0.136
Viral load before treatment, mean ± SD/median	832,595 ± 1,771,006/129,138	360,107 ± 669,913/131,396	593,964 ± 1,347,958/129,792	0.287
CD4 count before treatment, mean ± SD/median	265.44 ± 264.12/192	198.52 ± 179.12/192	231.64 ± 226.59/192	0.348

**Table 3:** Longitudinal serum amylase levels over two-year follow-up period stratified by treatment regimen.

Timepoint	INSTI (*n* = 49)	NNRTI/PI (*n* = 50)	Total (*n* = 99)	*p*-Value
Before treatment	78.71 ± 32.35/71	122.88 ± 42.06/112	101.02 ± 43.47/95	<0.0001
1 Year after treatment	69.44 ± 17.91/68	125.04 ± 41.17/119	97.52 ± 42.46/95	<0.0001
2 Years after treatment	68.44 ± 23.44/65	129.60 ± 37.97/117	99.33 ± 43.98/101	<0.0001

**Table 4:** Longitudinal liver enzyme levels (ALT/AST) over two-year follow-up period stratified by treatment regimen.

Variable	INSTI (*n* = 49)	NNRTI/PI (*n* = 50)	Total (*n* = 99)	*p*-Value
ALT—baseline	37.51 ± 32/29	26.98 ± 19/20	32.19 ± 26/24	0.051
ALT—1 year	28.58 ± 64.24/18	24.04 ± 19.19/19	26.26 ± 46.80/18	0.403
ALT—2 years	27.18 ± 25.08/19	27.84 ± 23.30/21	27.51 ± 24.08/20	0.75
AST—baseline	29.79 ± 28.51/26	33.96 ± 20.59/28	31.89 ± 24.79/27	0.106
AST—1 year	32.08 ± 73.32/20	26.24 ± 10.55/24	29.13 ± 51.94/22	0.011
AST—2 years	22.36 ± 8.35/20	28.64 ± 22.04/24	25.53 ± 16.94/22	0.056

**Table 5:** Longitudinal serum creatinine levels over two-year follow-up period stratified by treatment regimen.

Timepoint	INSTI (*n* = 49)	NNRTI/PI (*n* = 50)	Total (*n* = 99)	*p*-Value
Cr Baseline	0.84 ± 0.18/0.840	2.67 ± 14.48/0.625	1.77 ± 10.28/0.760	<0.0001
Cr 1 year after treatment	0.92 ± 0.205/0.960	1.62 ± 6.839/0.680	1.28 ± 4.851/0.800	<0.0001
Cr 2 years after treatment	0.91 ± 0.197/0.930	0.6668 ± 0.225/0.660	0.78 ± 0.244/0.800	<0.0001

### 3.4. Repeated-Measures Analysis

**Figure 1:**
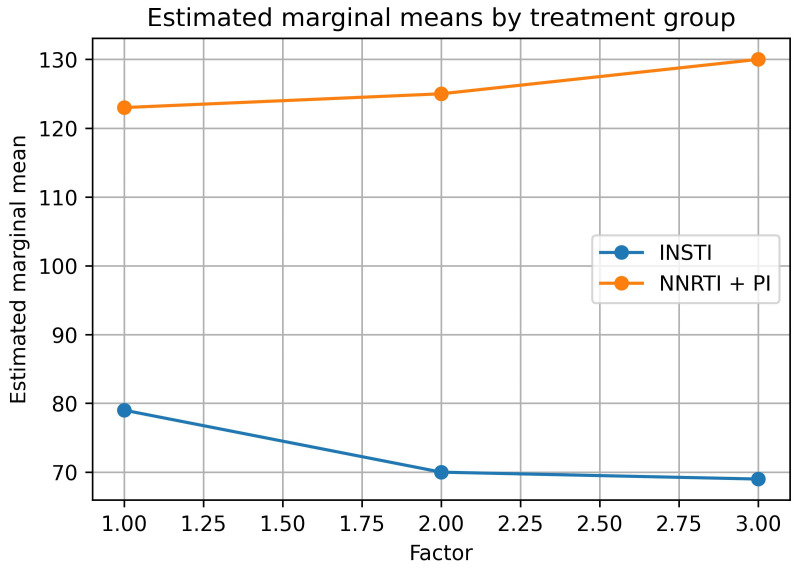
Mean serum amylase levels (±standard deviation) over two-year follow-up stratified by antiretroviral therapy regimen. The graph illustrates the divergent trajectories between treatment groups across the follow-up period. INSTI-based regimens (solid line, circles) showed stable or declining amylase levels, while NNRTI/PI-based regimens (dashed line, squares) maintained persistently elevated levels. Error bars represent ± 1 standard deviation. The significant linear time × treatment group interaction (*p* = 0.037) indicates that amylase trajectories differed significantly between regimens over the two-year period. Abbreviations: INSTI, integrase strand transfer inhibitor; NNRTI, non-nucleoside reverse transcriptase inhibitor; PI, protease inhibitor; U/L, units per liter.

Repeated-measures ANOVA revealed no significant overall change in serum amylase levels over time (*p* = 0.61) within each group individually. However, a trend-level interaction between time and treatment group was observed (*p* = 0.067), suggesting potential differences in the trajectory of amylase levels between regimens. Polynomial contrast analysis demonstrated a significant linear interaction between time and group (F (1,97) = 4.450, *p* = 0.037), indicating that amylase levels changed in opposite directions across groups—decreasing in the INSTI group while remaining stable or slightly increasing in the NNRTI/PI group (Figure 1).

These longitudinal findings remained meaningful despite baseline differences, as the direction of change over time differed significantly between treatment groups (Table 3).

### 3.5. Subgroup Analysis by Ethnicity

When stratified by ethnicity (Supplementary), baseline amylase levels varied across ethnic groups but consistently demonstrated lower values in INSTI-treated patients compared to NNRTI/PI-treated patients within each stratum. Ethiopian-origin patients showed the highest baseline amylase levels (overall mean 132 ± 46 U/L), followed by patients of other ethnicities (104 ± 41 U/L), ex-Soviet Union origin (83 ± 31 U/L), and Israeli-born patients (79 ± 28 U/L). The difference between treatment groups reached statistical significance in Israeli-born (*p* = 0.003) and ex-Soviet Union cohorts (*p* = 0.041), but not in Ethiopian (*p* = 0.539) or other ethnicity groups (*p* = 0.414), likely due to small subgroup sample sizes. Importantly, the direction of the treatment effect was consistent across all ethnic groups, with INSTI-based regimens associated with lower amylase levels regardless of ethnicity.

### 3.6. Correlation Analysis

Correlation analysis between baseline viral load and CD4 count demonstrated a significant inverse relationship in the NNRTI/PI group (r = –0.328, *p* = 0.020, Supplementary Table S1), consistent with expected HIV disease pathophysiology. This correlation was not significant in the overall cohort (r = –0.104, *p* = 0.304) or INSTI group (r = –0.088, *p* = 0.547). The lack of baseline differences in viral suppression rates (Table 2) and the persistence of group differences in amylase levels after achieving viral suppression suggest that the observed amylase patterns are primarily related to ART regimen class rather than HIV disease activity. Similarly, the stability of renal function in both groups throughout follow-up (Table 5, mean creatinine values within normal range) indicates that kidney function did not confound the observed amylase differences.

## 4. Discussion

Our findings suggest an association between INSTI-based antiretroviral regimens and more favorable serum amylase profiles compared with NNRTI/PI-based therapies. However, given the retrospective observational design, temporal bias, and demographic imbalances between treatment groups, these findings should be interpreted as hypothesis-generating rather than definitive evidence of superior pancreatic safety. The observed differences in amylase trajectories may reflect true drug class effects, temporal changes in clinical practice and patient populations, unmeasured demographic or genetic factors, or a combination of these influences. Prospective comparative studies with balanced baseline characteristics, longer follow-up periods, and assessment of clinical outcomes (including acute pancreatitis incidence) are needed to definitively establish whether INSTI-based regimens offer superior pancreatic safety compared to older-generation therapies [5,10,11,12].

No clinical pancreatitis was observed in either treatment group, and all observed variations in serum amylase were biochemical and asymptomatic, supporting the pancreatic safety of modern ART.

In the pre-HAART era, treatment for HIV primarily involved older NRTIs such as didanosine (ddI) and stavudine (d4T). These drugs were strongly linked to mitochondrial toxicity and a dose-dependent risk of acute pancreatitis [2,3].

Lamivudine, introduced later as a safer and more effective NRTI, was generally well-tolerated and widely used as part of combination antiretroviral therapy. Although rare, transient elevations in amylase have been reported. These enzyme elevations are usually asymptomatic and self-limited, likely associated with cumulative metabolic stress rather than direct toxicity [2,13].

With the advent of HAART, combining three or more drugs from at least two classes dramatically improved HIV survival but introduced new metabolic complications. NNRTIs such as efavirenz, which was introduced during this period, generally had a favorable metabolic profile but were occasionally associated with hyperamylasemia and pancreatitis [14].

Early HAART regimens often combined first-generation PIs such as indinavir (IDV) with older NRTIs like ddI and d4T. Such combinations resulted in markedly increased pancreatitis rates, attributed mainly to additive mitochondrial and metabolic stress, rather than direct PI toxicity [7,15]. Newer PIs such as lopinavir/ritonavir demonstrated an improved safety profile; while linked to hypertriglyceridemia and insulin resistance, clinical pancreatitis remained rare, and enzyme elevations were typically mild and linked to metabolic abnormalities [14,16,17].

The introduction of Truvada, a fixed-dose combination of tenofovir disoproxil fumarate (TDF) and emtricitabine (FTC), marked a new generation of NRTI therapy with a safer mitochondrial toxicity profile [18]. In published data, TDF/FTC was not consistently associated with elevated serum amylase or clinical pancreatitis. Most reports describe rare or isolated cases, typically when combined with didanosine or protease inhibitors, suggesting pharmacokinetic or metabolic interactions rather than a direct TDF/FTC effect [19]. In our dataset, patients treated with Truvada-containing regimens demonstrated mild, non-significant increases in mean serum amylase levels, particularly when co-administered with PIs, consistent with additive metabolic mechanisms proposed in prior literature.

The development of INSTIs, such as raltegravir (RAL), dolutegravir (DTG), and bictegravir (BIC), has revolutionized HIV therapy by providing high efficacy and improved tolerability, with a favorable metabolic profile compared with earlier regimens. INSTIs are now leading components in first-line ART worldwide. Safety summaries for INSTIs mainly describe gastrointestinal and neuropsychiatric side effects [20,21], and there are not enough data addressing changes in pancreatic enzymes or comparisons with older ART regimens in this regard.

In our study, INSTI-based regimens demonstrated a mild, non-significant decline in mean amylase values over time, without any cases of pancreatitis or clinically relevant enzyme elevations, confirming their favorable pancreatic safety profile.

Beyond drug class effects, we also examined clinical and demographic correlates of amylase behavior. The observed differences in amylase patterns appeared to be independent of viral suppression and immune recovery, as reflected by CD4^+^ cell counts In our study, a significant inverse correlation between viral load and CD4 count was observed in the NNRTI/PI group (r = –0.33, *p* = 0.02), as expected in people living with HIV, though this relationship was not significant in the overall cohort or INSTI sub-group. Previous studies, however, have described higher amylase levels among patients with advanced HIV disease and low CD4 counts, suggesting that immune suppression may contribute to subclinical pancreatic changes [22].

The absence of this relationship in our cohort likely reflects the fact that most participants had achieved viral suppression and immune reconstitution.

Although Ethiopian-origin patients were more prevalent in the NNRTI/PI group (≈60%) and Israeli-born and ex-Soviet-origin patients were more common in the INSTI group, subgroup analysis showed no significant association between ethnicity, risk category, renal function, or other demographic variables and serum amylase levels.

These demographic differences reflect the temporal distribution of patient enrollment, with NNRTI/PI-treated patients predominantly initiating therapy in the earlier period (2002–2015) and INSTI-treated patients predominantly initiating therapy in the later period (2016–2023), corresponding to evolution in HIV treatment guidelines and changes in the epidemiological characteristics of newly diagnosed HIV patients in Israel over this time period.

Repeated-measures ANOVA revealed no significant overall change in serum amylase levels over time when examined across both groups combined (within-subjects main effect of time: *p* = 0.61). However, polynomial contrast analysis demonstrated a significant linear time × treatment group interaction (F (1,97) = 4.450, *p* = 0.037), indicating that the trajectory of amylase changes over time differed significantly between treatment groups. Specifically, amylase levels decreased over time in the INSTI group (from 78.7 ± 32.3 U/L at baseline to 68.4 ± 23.4 U/L at two years) while remaining elevated or slightly increasing in the NNRTI/PI group (from 122.9 ± 42.1 U/L at baseline to 129.6 ± 38.0 U/L at two years), representing divergent trajectories over the two-year follow-up period (Figure 1). The between-subjects effect of treatment group was highly significant (*p* < 0.0001 at all time points), reflecting the consistently higher amylase levels in the NNRTI/PI group compared to the INSTI group throughout the entire observation period. These findings suggest differential effects of the ART class on amylase dynamics, though the observed differences must be interpreted in the context of baseline demographic imbalances and temporal bias as discussed in the Limitations section.

Overall, these findings indicate that the variation in amylase observed in this study is best explained by ART class effects rather than by demographic or immunologic factors.

Taken together, our findings align with current literature suggesting that the ART regimen class remains the dominant determinant of pancreatic enzyme behavior, independent of age, sex, or geography. INSTI-based regimens demonstrated a more stable biochemical profile and fewer metabolic disturbances, while PI/NNRTI-based therapies were associated with persistently higher amylase levels, consistent with indirect metabolic effects.

These results underscore the importance of ongoing biochemical monitoring in HIV patients receiving any antiretroviral regimen. While our observations are consistent with the hypothesis that INSTI-based regimens may offer metabolic advantages, definitive recommendations regarding preferential regimen selection for patients at risk for pancreatic complications await validation in prospective studies designed to address the limitations inherent in our retrospective observational design.

### Limitations

This study has several limitations that should be acknowledged.

First, as a retrospective observational study, causal relationships could not be established, and reliance on medical record data may have introduced minor documentation or timing biases. Second, the study sample was relatively small, and all participants were recruited from a single center, which may limit the statistical power and generalizability of the findings to broader populations. Third, only total serum amylase levels were available for analysis. Since amylase is produced by both the pancreas and salivary glands, and isoenzyme differentiation (pancreatic vs. salivary amylase) was not performed, it was not possible to determine the specific origin of the enzyme elevations observed. Fourth, lipase levels were not included because this parameter is not routinely measured in the clinic; therefore, a parallel assessment of other pancreatic enzymes could not be conducted. Fifth, all participants were treatment-naïve prior to ART initiation and remained within the same regimen class throughout follow-up, eliminating potential bias from prior ART.

Additionally, our study grouped NNRTI-based and PI-based regimens together for comparison against INSTI-based therapy. While this approach reflects real-world prescribing practices and addresses the clinically relevant question of modern versus older-generation ART, it does not allow us to distinguish potential differential effects between NNRTIs and PIs specifically. Future studies with larger sample sizes would be valuable to further characterize drug-class-specific metabolic effects, though we note that contemporary treatment guidelines have largely moved toward INSTI-based regimens as preferred first-line therapy regardless of this distinction, making the INSTI versus non-INSTI comparison the most clinically relevant for current practice. Also, our study design assessed drug class effects (INSTI-based versus NNRTI/PI-based regimens) rather than effects of individual drugs within each class. While this approach is appropriate for our sample size and directly addresses the clinically relevant question of modern versus older-generation ART, it does not allow identification of potential differential effects between specific individual drugs (e.g., efavirenz versus rilpivirine, or lopinavir/ritonavir versus atazanavir). Future studies with larger sample sizes specifically designed to compare individual drug effects would be valuable to further refine understanding of drug-specific metabolic impacts. Furthermore, significant imbalances in baseline demographic characteristics between treatment groups represent an additional fundamental limitation. The NNRTI/PI group had substantially higher proportions of Ethiopian-origin patients (60% vs. 8.2%) and patients from endemic geographical risk groups (66% vs. 6.1%), while the INSTI group had higher proportions of Israeli-born and ex-Soviet-origin patients and MSM. These demographic differences are not randomly distributed but are intrinsically linked to the temporal bias described above, as immigration patterns and HIV epidemic characteristics evolved over the 21-year study period. While our subgroup analyses stratified by ethnicity suggested that the pattern of lower amylase levels in INSTI-treated patients was generally consistent across ethnic groups, and correlation analyses showed no significant associations between ethnicity or risk group and amylase levels, we cannot definitively exclude the possibility that genetic, anthropometric, dietary, or socioeconomic factors associated with ethnicity or risk group partially contribute to the observed differences in amylase trajectories. Advanced adjusted analyses such as repeated-measures ANCOVA or mixed-effects models incorporating demographic covariates were not feasible given our limited sample size, which would result in overfitted models with unstable estimates.

## 5. Conclusions

The relationship between HIV, serum amylase levels, and HAART is complex and influenced by various factors, including the type of antiretroviral therapy, the duration of HIV infection, and the presence of other comorbid conditions. In this retrospective study of people living with HIV, no cases of clinical pancreatitis were observed, and all changes in serum amylase were mild and asymptomatic.

Patients receiving INSTI-based regimens maintained stable or slightly decreased amylase levels, whereas those treated with NNRTI/PI-based therapy showed persistently higher mean values throughout two years of follow-up.

These differences were independent of age, sex, renal function, and CD4 cell counts, indicating that ART drug class—rather than immunologic or demographic factors—was the principal determinant of enzyme variation. Our findings therefore suggest that INSTI-based antiretroviral regimens are associated with a favorable pancreatic and metabolic safety profile compared with NNRTI/PI-based therapies.

Continuous biochemical monitoring remains advisable, particularly for patients with additional metabolic risk factors or prolonged exposure to PI- or NNRTI-containing combinations. Further prospective studies are warranted to elucidate the underlying mechanisms and to assess clinical significance, including any potential implications for pancreatic health in long-term ART.

## Data Availability

Data are available from the corresponding author upon reasonable request.

## Supplementary Materials

The following supporting information can be downloaded at: https://www.mdpi.com/article/10.3390/v18010045/s1, Table S1: Correlation Analysis Between Baseline Viral Load and CD4 Count; Table S2: Detailed Demographic Comparisons by Ethnicity and Treatment; Table S3: Comparison of Column Proportions—Significant Pairwise Differences; Table S4: Independent Samples *t*-tests for Continuous Variables; Table S5: Mann-Whitney U Tests for Non-Normally Distributed Variables; Table S6: Descriptive Statistics—Complete Dataset; Table S7: Study population and exclusions; Table S8: Baseline Serum Amylase Levels Stratified by Ethnicity and Treatment Regimen.

## Abbreviations

AIDS	Acquired Immune Deficiency Syndrome
ALT	Alanine Aminotransferase
ANOVA	Analysis of Variance
ART	Antiretroviral Therapy
AST	Aspartate Aminotransferase
BIC	Bictegravir
BIC/FTC/TAF	Bictegravir/Emtricitabine/Tenofovir Alafenamide
CD4	Cluster of Differentiation 4 (CD4^+^ T-lymphocytes)
Cr	Creatinine
d4T	Stavudine
ddI	Didanosine
DTG	Dolutegravir
END-GEO	Endemic Geographical Risk Group
FTC	Emtricitabine
GI	Gastrointestinal
HAART	Highly Active Antiretroviral Therapy
HCV	Hepatitis C Virus
HIV	Human Immunodeficiency Virus
IBD	Inflammatory Bowel Disease
IDV	Indinavir
IQR	Interquartile Range
INSTI	Integrase Strand Transfer Inhibitor
IVDU	Intravenous Drug Use
MSM	Men Who Have Sex With Men
ND	Not Detectable (ND < 50 copies/mL)
NNRTI	Non-Nucleoside Reverse Transcriptase Inhibitor
NRTI	Nucleoside Reverse Transcriptase Inhibitor
PI	Protease Inhibitor
PLWH	People Living With HIV
RAL	Raltegravir
RNA	Ribonucleic Acid (HIV RNA)
SD	Standard Deviation
SPSS	Statistical Package for the Social Sciences
TAF	Tenofovir Alafenamide
TDF	Tenofovir Disoproxil Fumarate
VL	Viral Load
