# Supplementary material for: Trends in Serum Amylase Levels in People Living with HIV: A Comparison Between INSTI and NNRTI/PI-Based Regimens
Source: Viruses. 2025 Dec 27;18(1):45. doi: 10.3390/v18010045 (PMC12846545; doi:10.3390/v18010045)
Supplement: Supplementary file 1 [file viruses-18-00045-s001.zip › viruses-4026584-supplementary.pdf]

Supplementary File

Supplementary Table S1: Correlation Analysis Between Baseline Viral Load and CD4 Count

Group	Variables	Pearson Correlation (r)	Significance (2-tailed)	N
**Overall**	VL baseline vs. CD4 baseline	-0.104	0.304	99
**INSTI**	VL baseline vs. CD4 baseline	-0.088	0.547	49
**NNRTI/PI**	VL baseline vs. CD4 baseline	-0.328*	0.020	50

*p < 0.05

Note: A significant inverse correlation between baseline viral load and CD4 count was observed in the NNRTI/PI group (r = -0.328, p = 0.020), consistent with expected HIV disease pathophysiology. The correlation was weaker and non-significant in the INSTI group, though this may reflect the smaller sample size or different disease characteristics at presentation in the later time period when INSTI therapy became available.

Supplementary Table S2: Detailed Demographic Comparisons by Ethnicity and Treatment Group

Ethiopian-Origin Patients (n=34)

Variable	INSTI (n=4)	NNRTI/PI (n=30)	Total (n=34)	p-value
**Sex**				0.347
Male	1 (25.0%)	15 (50.0%)	16 (47.1%)	
Female	3 (75.0%)	15 (50.0%)	18 (52.9%)	
**Risk Group**				0.005*
END-GEO	3 (75.0%)	30 (100.0%)	33 (97.1%)	
MSM	0 (0%)	0 (0%)	0 (0%)	
IVDU	0 (0%)	0 (0%)	0 (0%)	
Other	1 (25.0%)	0 (0%)	1 (2.9%)	
**VL 1 year after treatment**				NA
ND + <50	4 (100%)	30 (100%)	34 (100%)	
**Comorbidities**				0.947
None	4 (100%)	27 (90.0%)	31 (91.2%)	
Present	0 (0%)	3 (10.0%)	3 (8.8%)	

Variable	INSTI (n=4)	NNRTI/PI (n=30)	Total (n=34)	p-value
**Smoking**				0.278
Yes	0 (0%)	7 (23.3%)	7 (20.6%)	
No	4 (100%)	23 (76.7%)	27 (79.4%)	

*p < 0.05; NA = not applicable (no variation)

Israeli-Born Patients (n=31)

Variable	INSTI (n=21)	NNRTI/PI (n=10)	Total (n=31)	p-value
**Sex**				0.141
Male	9 (42.9%)	3 (30.0%)	12 (38.7%)	
Female	12 (57.1%)	7 (70.0%)	19 (61.3%)	
**Risk Group**				0.328
END-GEO	1 (4.8%)	1 (10.0%)	2 (6.5%)	
MSM	13 (61.9%)	6 (60.0%)	19 (61.3%)	
IVDU	0 (0%)	0 (0%)	0 (0%)	
Other	7 (33.3%)	3 (30.0%)	10 (32.3%)	
**VL 1 year after treatment**				0.312
ND + <50	19 (90.5%)	9 (90.0%)	28 (90.3%)	
60-184	2 (9.5%)	1 (10.0%)	3 (9.7%)	
**Comorbidities**				0.427
None	18 (85.7%)	9 (90.0%)	27 (87.1%)	
Present	3 (14.3%)	1 (10.0%)	4 (12.9%)	
**Smoking**				0.919
Yes	8 (38.1%)	4 (40.0%)	12 (38.7%)	
No	13 (61.9%)	6 (60.0%)	19 (61.3%)	

Ex-Soviet Union Origin Patients (n=22)

Variable	INSTI (n=19)	NNRTI/PI (n=3)	Total (n=22)	p-value
**Sex**				0.907

Variable	INSTI (n=19)	NNRTI/PI (n=3)	Total (n=22)	p-value
Male	12 (63.2%)	2 (66.7%)	14 (63.6%)	
Female	7 (36.8%)	1 (33.3%)	8 (36.4%)	
**Risk Group**				0.010*
END-GEO	0 (0%)	0 (0%)	0 (0%)	
MSM	2 (10.5%)	0 (0%)	2 (9.1%)	
IVDU	3 (15.8%)	1 (33.3%)	4 (18.2%)	
Other	14 (73.7%)	2 (66.7%)	16 (72.7%)	
**VL 1 year after treatment**				0.796
ND + <50	19 (100%)	2 (66.7%)	21 (95.5%)	
184	0 (0%)	1 (33.3%)	1 (4.5%)	
**Comorbidities**				0.680
None	14 (73.7%)	3 (100%)	17 (77.3%)	
Liver disease	3 (15.8%)	0 (0%)	3 (13.6%)	
Pancreatitis + Liver	1 (5.3%)	0 (0%)	1 (4.5%)	
Alcohol + Liver	1 (5.3%)	0 (0%)	1 (4.5%)	
**Smoking**				0.427
Yes	8 (42.1%)	2 (66.7%)	10 (45.5%)	
No	11 (57.9%)	1 (33.3%)	12 (54.5%)	

*p < 0.05

Other Ethnicity Patients (n=12)

Variable	INSTI (n=5)	NNRTI/PI (n=7)	Total (n=12)	p-value
**Sex**				0.408
Male	4 (80.0%)	4 (57.1%)	8 (66.7%)	
Female	1 (20.0%)	3 (42.9%)	4 (33.3%)	
**Risk Group**				0.216
END-GEO	2 (40.0%)	2 (28.6%)	4 (33.3%)	

**Variable**	**INSTI (n=5)**	**NNRTI/PI (n=7)**	**Total (n=12)**	**p-value**
MSM	0 (0%)	0 (0%)	0 (0%)	
IVDU	1 (20.0%)	2 (28.6%)	3 (25.0%)	
Other	2 (40.0%)	3 (42.9%)	5 (41.7%)	
**VL 1 year after treatment**				NA
ND + <50	7 (100%)	7 (100%)	12 (100%)	
**Comorbidities**				0.345
None	6 (85.7%)	7 (100%)	11 (91.7%)	
GI disease	0 (0%)	1 (14.3%)	1 (8.3%)	
**Smoking**				0.793
Yes	1 (20.0%)	3 (42.9%)	4 (33.3%)	
No	6 (85.7%)	6 (85.7%)	10 (83.3%)	

Supplementary Table S3: Comparison of Column Proportions - Significant Pairwise Differences

Origin (Ethnicity)

**Comparison**	**Group A (INSTI)**	**Group B (NNRTI/PI)**	**Adjusted p-value**
Ethiopian	8.2%	60.0%	< 0.001*
Israeli	42.9%	20.0%	0.014*
Ex-Soviet Union	38.8%	6.0%	< 0.001*

*Significant at p < 0.05 (Bonferroni-adjusted for multiple pairwise comparisons)

Interpretation: Ethiopian-origin patients were significantly overrepresented in the NNRTI/PI group, while Israeli-born and ex-Soviet origin patients were significantly overrepresented in the INSTI group. These differences reflect temporal changes in the HIV epidemic in Israel, with Ethiopian immigrants representing a larger proportion of newly diagnosed HIV cases in the earlier period (2002-2015) and Israeli-born/ex-Soviet patients representing larger proportions in the later period (2016-2023).

Risk Group

**Comparison**	**Group A (INSTI)**	**Group B (NNRTI/PI)**	**Adjusted p-value**
END-GEO	6.1%	66.0%	< 0.001*

Comparison	Group A (INSTI)	Group B (NNRTI/PI)	Adjusted p-value
MSM	34.7%	12.0%	0.008*
Other	51.0%	18.0%	0.001*

*Significant at $p < 0.05$ (Bonferroni-adjusted for multiple pairwise comparisons)

Interpretation: Patients from endemic geographical regions were significantly overrepresented in the NNRTI/PI group, while MSM were significantly overrepresented in the INSTI group. This pattern reflects evolving HIV epidemic characteristics in Israel over the study period.

Supplementary Table S4: Independent Samples t-Tests for Continuous Variables

Variable	INSTI (n=49) Mean ± SD	NNRTI/PI (n=50) Mean ± SD	Mean Difference	95% CI	t	df	p-value
**Age at treatment initiation (years)**	37.7 ± 12.3	42.0 ± 16.3	-4.37	[-10.14, 1.40]	-1.502	97	0.136
**Baseline VL (copies/mL)**	832,595 ± 1,771,006	360,107 ± 669,913	472,488	[-427,359, 1,372,335]	1.062	97	0.305†
**Baseline CD4 (cells/µL)**	265.4 ± 264.1	198.5 ± 179.1	66.92	[-18.82, 152.66]	Not reported	97	0.348
**Baseline Amylase (U/L)**	78.7 ± 32.3	122.9 ± 42.1	-44.2	[-58.7, -29.6]	5.765	97	<0.001*
**1-year Amylase (U/L)**	69.4 ± 18.9	125.0 ± 41.2	-55.6	[-68.4, -42.8]	7.598	97	<0.001*
**2-year Amylase (U/L)**	68.4 ± 23.4	129.6 ± 38.0	-61.2	[-73.1, -49.2]	7.871	97	<0.001*
**Baseline ALT (U/L)**	37.5 ± 32.0	27.0 ± 19.0	10.5	[-0.2, 21.3]	1.950	97	0.051
**Baseline AST (U/L)**	29.8 ± 28.5	34.0 ± 20.6	-4.2	[-13.6, 5.3]	-1.618	97	0.106
**Baseline Creatinine (mg/dL)**	0.84 ± 0.18	2.67 ± 14.48	-1.83	[-4.97, 1.31]	Not reliable	97	<0.001‡

*p < 0.001; †Mann-Whitney U test used due to non-normal distribution; ‡Due to extreme outliers in NNRTI/PI group

Note: For viral load, Mann-Whitney U test was used (U = 1073.0, Z = -1.064, p = 0.287) due to significant positive skew. For CD4 count, Mann-Whitney U test was used (U = 1091.0, Z = -0.938, p = 0.348). The large variance in baseline creatinine is due to 2-3 patients with moderately elevated values (1.4-2.5 mg/dL) representing mild chronic kidney disease that did not meet exclusion criteria.

Supplementary Table S5: Mann-Whitney U Tests for Non-Normally Distributed Variables

Variable	INSTI (n=49) Median [IQR]	NNRTI/PI (n=50) Median [IQR]	Mann-Whitney U	Wilcoxon W	Z	p-value
**Baseline VL**	129,138 [48,000–1,000,000]	131,396 [32,234–301,803]	1073.0	2348.0	-1.064	0.287
**Baseline CD4**	192 [54–360]	192 [77–248]	1091.0	2366.0	-0.938	0.348
**Baseline Amylase**	71 [61–92]	112 [96–146]	425.0	1650.0	-5.600	<0.001*
**1-year Amylase**	68 [56–81]	119 [108–140]	139.5	1364.5	-7.598	<0.001*
**2-year Amylase**	65 [54–82]	117 [109–137]	100.5	1325.5	-7.871	<0.001*
**Baseline Creatinine**	0.84 [0.77–0.95]	0.625 [0.48–0.75]	494.0	1769.0	-5.118	<0.001*

*p < 0.001

Note: Despite the statistically significant difference in creatinine, median values in both groups remained within normal range. The significant difference is driven by higher variability in the NNRTI/PI group rather than clinically meaningful renal dysfunction.

Supplementary Table S6: Descriptive Statistics - Complete Dataset

Variable	INSTI (n=49)	NNRTI/PI (n=50)	Total (n=99)
**Age (years)**			
Mean ± SD	37.7 ± 12.3	42.0 ± 16.3	39.9 ± 14.6
Median	37	41	37
Range	13-63	22-74	13-74

Variable	INSTI (n=49)	NNRTI/PI (n=50)	Total (n=99)
25th-75th percentile	32-45	33-55	32-51
**Baseline Viral Load (copies/mL)**			
Mean ± SD	832,595 ± 1,771,006	360,107 ± 669,913	593,965 ± 1,347,959
Median	129,138	131,396	129,792
Range	1083-10,000,000	1005-3,244,366	1005-10,000,000
**Baseline CD4 (cells/µL)**			
Mean ± SD	265.4 ± 264.1	198.5 ± 179.1	231.6 ± 226.6
Median	192	192	192
Range	0-1073	5-881	0-1073
**Baseline Amylase (U/L)**			
Mean ± SD	78.7 ± 32.3	122.9 ± 42.1	101.0 ± 43.5
Median	71	112	95
Range	25-171	50-289	25-289
25th-75th percentile	61-92	96-146	70-132
**1-Year Amylase (U/L)**			
Mean ± SD	69.4 ± 18.9	125.0 ± 41.2	97.5 ± 42.5
Median	68	119	95
Range	24-119	26-309	24-309
**2-Year Amylase (U/L)**			
Mean ± SD	68.4 ± 23.4	129.6 ± 38.0	99.3 ± 44.0
Median	65	117	101
Range	24-146	55-290	24-290

Supplementary Table S7 : Study population and exclusions

Category	n	% of total (N=1,202)
**Total patients assessed**	**1,202**	**100%**
**Excluded patients (total)**	**1,103**	**91.8%**
1. Patients without continuous 2-year follow-up	617	51.3%
2. No continuous treatment for 2 years	121	10.1%
3. Non-naïve patients	75	6.2%
4. Missing data (treatment initiated elsewhere / missing initiation data)	19	1.6%
5. No consecutive annual blood tests	26	2.2%
6. Died during the 2-year period	10	0.8%
7. Complex treatment / change in treatment group during 2 years	113	9.4%
**Included in final analysis**	**99**	**8.2%**

Supplementary Table S8 : Baseline Serum Amylase Levels Stratified by Ethnicity and Treatment Regimen

Ethnicity	Treatment	n	Mean ± SD (U/L)	Median [IQR] (U/L)	Range (U/L)	p-value*
**Ethiopian (n = 34)**	INSTI	4	118 ± 41	124 [89–147]	63–161	0.539
	NNRTI / PI	30	134 ± 46	132 [108–148]	69–289	
	**Total**	**34**	**132 ± 46**	**132 [108–148]**	**63–289**	
**Israeli-born (n = 31)**	INSTI	21	69 ± 25	70 [53–79]	29–138	**0.003†**
	NNRTI / PI	10	99 ± 20	108 [82–111]	70–134	
	**Total**	**31**	**79 ± 28**	**76 [61–101]**	**29–138**	
**Ex-Soviet Union (n = 22)**	INSTI	19	77 ± 28	73 [61–92]	25–159	**0.041†**
	NNRTI / PI	3	114 ± 32	108 [86–149]	86–149	
	**Total**	**22**	**83 ± 31**	**79 [62–95]**	**25–159**	

Ethnicity	Treatment	n	Mean ± SD (U/L)	Median [IQR] (U/L)	Range (U/L)	p-value*
**Other ethnicity (n = 12)**	INSTI	5	92 ± 47	69 [65–100]	57–171	0.414
	NNRTI / PI	7	113 ± 37	101 [96–146]	50–156	
	**Total**	**12**	**104 ± 41**	**100 [67–145]**	**50–171**	
**All ethnicities (n = 99)**	INSTI	49	79 ± 32	71 [61–92]	25–171	**<0.001†**
	NNRTI / PI	50	123 ± 42	112 [96–146]	50–289	
	**Total**	**99**	**101 ± 44**	**95 [70–132]**	**25–289**	

Data are presented as mean ± standard deviation, median with interquartile range [IQR], and range (minimum–maximum).

*P-values from independent samples t-test (for normally distributed data) or Mann-Whitney U test (for non-normally distributed data) comparing INSTI vs. NNRTI/PI groups within each ethnic stratum.

†Statistically significant at $p < 0.05$.

Note: Among Ethiopian-origin patients, the small sample size in the INSTI group (n=4) limits statistical power. Despite higher mean amylase levels in both Ethiopian subgroups compared to other ethnicities, the pattern of lower amylase in INSTI-treated patients compared to NNRTI/PI-treated patients was consistent across all ethnic groups, though this difference reached statistical significance only in the Israeli-born and Ex-Soviet Union cohorts due to larger sample sizes.
